# Dual pathways to endochondral osteoblasts: a novel chondrocyte-derived osteoprogenitor cell identified in hypertrophic cartilage

**DOI:** 10.1242/bio.201411031

**Published:** 2015-04-16

**Authors:** Jung Park, Matthias Gebhardt, Svitlana Golovchenko, Francesc Perez-Branguli, Takako Hattori, Christine Hartmann, Xin Zhou, Benoit deCrombrugghe, Michael Stock, Holm Schneider, Klaus von der Mark

**Affiliations:** 1 Dept. Exp. Medicine I, Nikolaus-Fiebiger Center of Molecular Medicine, University of Erlangen-Nuremberg, 91054 Erlangen, Germany; 2 Department of Pediatrics, Division of Molecular Pediatrics, University of Erlangen-Nuremberg, 91054 Erlangen, Germany; 3 Junior Research Group III, Nikolaus-Fiebiger Center of Molecular Medicine, University Hospital, 91054 Erlangen, Germany; 4 Dept. of Biochemistry and Molecular Dentistry, Okayama University Graduate School of Medicine, Dentistry, and Pharmaceutical Sciences, Okayama City,700-8525, Japan; 5 Dept. of Bone- and Skeletal Research, Institute of Experimental Musculoskeletal Medicine (IEMM), University Hospital Muenster, 48149 Muenster, Germany; 6 Dept. Genetics, MDAnderson Cancer Center, Houston, TX 77030, USA; 7 Dept. Internal Medicine III, University Hospital Erlangen, D-91054 Erlangen, Germany

**Keywords:** Hypertrophic chondrocyte, Osteoprogenitor cell, Transdifferentiation, Trabecular osteoblast

## Abstract

According to the general understanding, the chondrocyte lineage terminates with the elimination of late hypertrophic cells by apoptosis in the growth plate. However, recent cell tracking studies have shown that murine hypertrophic chondrocytes can survive beyond “terminal” differentiation and give rise to a progeny of osteoblasts participating in endochondral bone formation. The question how chondrocytes convert into osteoblasts, however, remained open. Following the cell fate of hypertrophic chondrocytes by genetic lineage tracing using *BACCol10;Cre* induced *YFP*-reporter gene expression we show that a progeny of Col10Cre-reporter labelled osteoprogenitor cells and osteoblasts appears in the primary spongiosa and participates – depending on the developmental stage – substantially in trabecular, endosteal, and cortical bone formation. YFP^+^ trabecular and endosteal cells isolated by FACS expressed Col1a1, osteocalcin and runx2, thus confirming their osteogenic phenotype. In searching for transitory cells between hypertrophic chondrocytes and trabecular osteoblasts we identified by confocal microscopy a novel, small YFP^+^Osx^+^ cell type with mitotic activity in the lower hypertrophic zone at the chondro-osseous junction. When isolated from growth plates by fractional enzymatic digestion, these cells termed CDOP (chondrocyte-derived osteoprogenitor) cells expressed bone typical genes and differentiated into osteoblasts *in vitro*. We propose the Col10Cre-labeled CDOP cells mark the initiation point of a second pathway giving rise to endochondral osteoblasts, alternative to perichondrium derived osteoprogenitor cells. These findings add to current concepts of chondrocyte-osteocyte lineages and give new insight into the complex cartilage-bone transition process in the growth plate.

## INTRODUCTION

Longitudinal bone growth in vertebrates is achieved by continuous, transient apposition of cartilaginous mass in the growth plates and subsequent substitution of cartilage by bone marrow and bone, a process summarized as endochondral ossification. The dynamic propulsion of the growth plate towards the epiphyses is achieved by a burst of chondrocyte proliferation, followed by differentiation into prehypertrophic, early and late (“terminally differentiated”) hypertrophic chondrocytes, associated with a dramatic increase in cell size and the deposition of a transient, calcified cartilage matrix (for reviews see Ballock and O'Keefe, 2003; Mackie et al., 2008; Provot and Schipani, 2005). The process is regulated by several transcription factors such as Sox9, Runx2 or FOXA (Lefebvre and Smits, 2005; Long and Ornitz, 2013; Takeda et al., 2001) and numerous growth factors and their receptors including IGF, FGFs BMPs, PTHRP (Kronenberg, 2006), IHH (Wuelling and Vortkamp, 2011) and Wnt factors (Day and Yang, 2008; Hartmann, 2007) (for reviews, see Long and Ornitz, 2013; Mackie et al., 2008).

Concomitantly with the formation of hypertrophic cartilage in the diaphysis, mesenchymal cells in the surrounding perichondrium, in particular in the groove of Ranvier (Langenskiöld, 1998), differentiate into osteoprogenitor cells, forming the periosteum and subsequently the calcified cortical bone shaft of long bones (Bianco et al., 1998). Simultaneously, hypertrophic cartilage in the center of the diaphysis is resorbed by osteoclasts invading together with osteoprogenitors and other bone marrow cells through capillaries from the periosteum, forming the primary ossification center. The resorption process at the chondro-osseous junction interface leaves behind spicules of calcified cartilage. Those are ensheathed by osteoid produced by osteoblasts, thus forming the bone trabeculae of the primary spongiosa. Based on previous lineage tracing experiments it was concluded that the spongiosa forming osteoblasts originate from invading, periosteum-derived osteoprogenitor cells (Colnot et al., 2004; Maes et al., 2010).

The cell fate of hypertrophic chondrocytes at the chondro-osseous junction has been an issue of debate for a long time (Shapiro et al., 2005). Hypertrophic chondrocytes are extremely large cells with a 7–10 fold volume increase compared to proliferating chondrocytes. They are actively involved in the regulation of cartilage calcification and remodeling and vascular invasion. They produce a unique collagen, type X collagen (*Col10a1*), but express also a number of genes typical for bone cells [*alkaline phosphatase*, *osteopontin*, *BSP *(*bone sialo protein*), *Osx*, *Bglap *(*osteocalcin*, *ocn*) and *Runx2*] (Lefebvre and Smits, 2005). Late hypertrophic chondrocytes at the cartilage-bone marrow interface secrete vascular endothelial growth factor (VEGF) to attract invading endothelial cells (Gerber et al., 1999; Zelzer et al., 2004) and matrix metalloproteinases, mostly MMP13 (Inada et al., 2004). Numerous studies indicated that the terminally differentiated hypertrophic chondrocytes die by apoptosis or other forms of cell death shortly before invasion of endothelial cells (Aizawa et al., 1997; Farnum and Wilsman, 1987; Farnum and Wilsman, 1989; Gibson, 1998; Gibson et al., 1995; Zenmyo et al., 1996). The molecular mechanism of chondrocyte death and the relative proportion of chondrocytes undergoing apoptosis during endochondral ossification are, however, under debate (Horton et al., 1998).

An alternatively discussed cell fate of hypertrophic chondrocytes is their conversion into osteoblasts. In a number of experimental *in vivo* and *in vitro* systems evidence for a transdifferentiation process of chondrocytes into osteoblasts has been reported (Descalzi Cancedda et al., 1992; Enishi et al., 2014; Gentili et al., 1993; Holmbeck et al., 2003; Kirsch et al., 1992; Moskalewski and Malejczyk, 1989; Serafini et al., 2014; Silbermann et al., 1983; Thesingh et al., 1991). This alternative fate has for a long time been questioned, but three recent publications have provided convincing experimental evidence for a continuous chondrocyte-to-osteoblast lineage on the basis of a cell specific, tamoxifen inducible genetic recombination approach (Yang et al., 2014a; Yang et al., 2014b; Zhou et al., 2014).

Here we report on a molecular genetic approach to elucidate the cell fate of hypertrophic chondrocytes *in vivo by* performing lineage tracing experiments using *BAC-Col10-Cre* deleter mice to activate *ROSA26 LacZ* and *ROSA26 YFP* reporter genes in hypertrophic chondrocytes. The *BAC-Col10-Cre* mouse lines used in this study have previously been shown to express *Cre* specifically in hypertrophic chondrocytes, but not in other skeleton-related cells (Gebhard et al., 2008; Golovchenko et al., 2013). The results of our cell fate analysis are consistent with those of the recent reports (Yang et al., 2014a; Yang et al., 2014b; Zhou et al., 2014). We show that at early embryonic stages the *BAC-Col10-Cre;ROSA26* driven *LacZ* and *YFP* expression is restricted to hypertrophic chondrocytes before the formation of the primary ossification center. With the onset of bone marrow formation, however, we observed a substantial number of osteoblasts associated with subchondral trabeculae, endosteal and cortical bone that stained positive for β-gal or YFP. This indicates that these cells originated from Col10a1-expressing chondrocytes. In searching for the mechanism of chondrocyte-osteoblast conversion, we identified by confocal microscopy a small, proliferating Osx^+^YFP^+^ cell in the lower hypertrophic zone close to the chondro-osseous junction. We isolated these cells from growth plates of Col10CreYFP^+^ long bones and show that they express stem cell and osteoblast markers and differentiate into osteoblasts *in vitro*. Thus, our findings indicate that the terminal hypertrophic chondrocyte is not inevitably eliminated by cell death, but has the option to convert into a mitotically active new cell type with the potential to differentiate into osteoblasts.

## MATERIALS AND METHODS

### Transgenic mouse lines

Males from two independent BACCol10Cre deleter mouse lines (#1421 and #1465, described in Gebhard et al., 2008) were mated to *ROSA26^fl/fl^LacZ (R26R)* (Soriano, 1999) and *ROSA26^fl/fl^ YFP* (JAX: *B6.129X1-Gt(ROSA)26Sortm1(EYFP)Cos/J*) females. Both lines produced identical results. The offspring was genotyped by PCR, X-gal staining or by YFP-fluorescence analysis of digits, respectively as described below.

The care and use of the experimental animals complied with the institutional and German animal welfare laws, guidelines and policies. Animal experiments were carried out under the license TS10/12 NFZ.

### Immunofluorescence and immunohistochemistry

Paraffin and unfixed frozen sections of long bones, ribs and vertebrae from *Col10Cre;YFP* mice were predigested with hyaluronidase (Roche) and EDTA and stained with antibodies as described previously (Golovchenko et al., 2013; Hattori et al., 2010). Endosteal cells were cultured on fibronectin coated chamber slides prior to staining.

Immunolabeling was performed using the following antibodies: rat anti collagen I (kindly provided by Dr. Takako Sasaki; 1:250);, rabbit anti Col 1 (1:200; Abcam #21286), osterix (1:200; Abcam # 22552), CD 31/PECAM (1:500; Abcam #28364), osteocalcin (1:100; Takara, mOC 1-20) all rabbit; as well as chicken anti GFP (Abcam #13970, 1:250). Isotype-matched non-immunoglobulins for rat and rabbits were used as controls. Sections were counterstained with Cy2, Cy3 and Cy5 conjugated goat antibodies and Hoechst 33342 or DAPI for nuclear staining. Fluorescence images were viewed under a Zeiss Axiophot microscope using the Openlab program (Zeiss). For paraffin sections, bones from X-gal-stained *Col10Cre;R26R* or *Col10Cre;YFP^+^* mice were decalcified in EDTA and embedded in paraffin as described (Gebhard et al., 2007; Gebhard et al., 2008). X-gal stained sections were counterstained with eosin. Osterix was stained on paraffin sections with anti osx (1:500; Abcam), followed by AP conjugated goat anti rabbit antibody (1:100, BioRad) and Fast Red color substrate (Dako). X-gal staining was performed as described previously (Gebhard et al., 2007; Hattori et al., 2010). Alizarin red staining was performed as described previously (Golovchenko et al., 2013) with 1% Alizarin red, pH 4,2.

### BrdU incorporation

Pregnant *Col10Cre;YFP^+^* females were injected intraperitoneally with 200 µl BrdU at day E19. Tibiae and femorae from YFP^+^ newborns were fixed in 4% paraformaldehyde for 1 h, embedded in 4% agarose and 25 µm Vibratome sections were cut for confocal microscopy. Tissue was blocked with 2% BSA for 1 h and stained for immunofluorescence analysis with rabbit anti BrdU (e-Bioscience), chick anti GFP antibodies (Abcam), and DAPI.

### Confocal microscopy

**Fig. 5.**
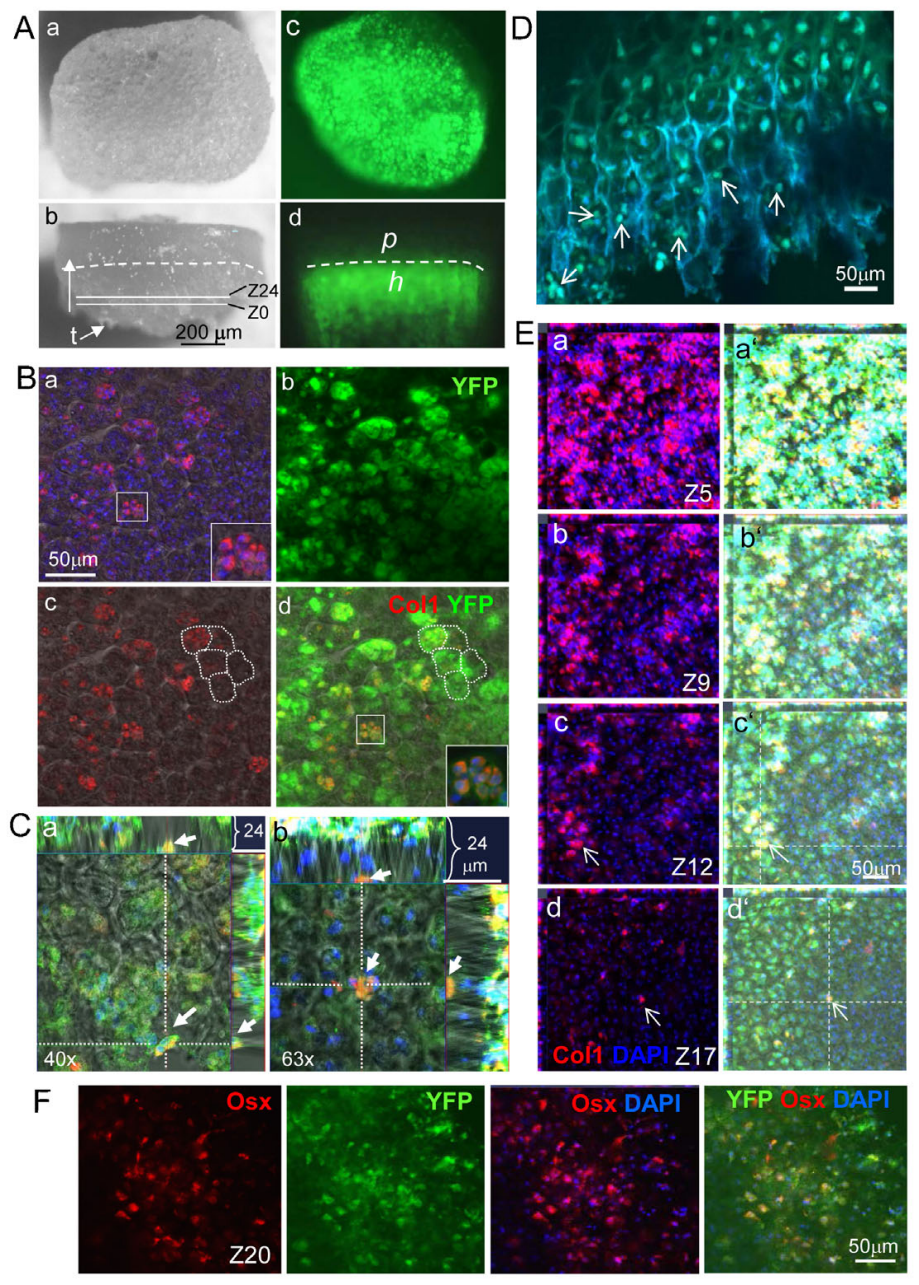
Identification of chondrocyte-derived osteoprogenitor (CDOP) cells in the growth plate by confocal microscopy. (A) Vertical (a,c) and lateral (b,d) view on growth plates, prepared by microdissection from a P5 *Col10Cre YFP^+^* tibia. The bone collar and the trabecular meshwork were removed from the cartilaginous part with a fine scalpel, but some trabeculae ‘t’ remain attached (b). Z0 and Z24 indicate the upper and lower limits of the scanned z-stacks. (b,d) The dashed line demarcates the border between the proliferating (p) and hypertrophic (h) zones, which was examined by confocal laser scanning microscopy. (c,d) Cre- induced YFP fluorescence. (B) Vertical view at the terminal zone of hypertrophic cartilage at the bone marrow interface in the proximal growth plate of a P5 *Col10CreYFP^+^* tibia by confocal laser scanning microscopy. A series of 22 to 24 z-stacked layers of 1 µm distance were photographed, each 100 nm thick, covering together 22–24 µm of the terminal hypertrophic zone (for orientation see also schematic supplementary material Fig. S4). Double staining for Col1 (a,c,d) and YFP (b,d) revealed numerous small Col1^+^YFP^+^ cells with a diameter of 4–6 µm, in the lowest layer of hypertrophic chondrocytes [lacunar walls delineated by white dotted lines in c,d as visualized by underlaying phase contrast (a,c,d)]. Islets in the bottom right corner in a,d: higher magnification of the cell cluster in the small inlets. (C) Positioning of selected small Col1^+^YFP^+^ cells (orange) along the x- and y-axis (main fields in a,b) in the chondro-osseous junction; the spongiosa is marked by a strong YFP fluorescence. The upper and right margins in a,b show the position of two individual YFP^+^Col1^+^ cells along the z-axis in the interface between hypertrophic cartilage and spongiosa, viewed from the x- or y-axis. (D) Confocal microscopy of sagittal vibratome sections (50 µm thick) of *Col10CreYFP^+^* tibia revealed small YFP^+^ cells (∼5 µm) (examples marked by arrows) at the chondro-osseous junction. (E) Localization of small Col1^+^YFP^+^ cells in 4 out of 22 stacks in the lower hypertrophic zone of a P5 *Col10CreYFP^+^* tibia chondro-osseous junction by confocal microscopy. The small Col1^+^YFP^+^ cells are preferentially seen in deeper levels (Z17, see arrows in d), while towards to the upper levels approaching the trabecular zone (Z12–Z1) Col1^+^YFP^+^ cells become larger (see arrow in c), and their number increases. (a'–d') The same images as those shown in a–d merged with the green YFP channel. (F) Localization of Osx^+^YFP^+^ cells in the lowest hypertrophic zone (Z20) of the growth plate by confocal microscopy. (a–d) Images show different channels of the same area, stained as indicated.

Growth plates from femora, tibiae and humeri of P5–P7 *Col10CreYFP^+^* mice and *YFP*^−^littermates were prepared under the stereomicroscope by microdissection and cleaned from periosteal bone collar and most of the spongy bone attached to the growth plate (see Fig. 5A). For confocal microscopy, growth plates were fixed for 30 min at 4°C in 4% paraformaldehyde, treated with testicular hyaluronidase (1 mg/ml) for 20 min, 0.5 M ETDA pH 7.5 for 30 min, and stained with antibodies after blocking with 2% BSA. Stained growth plates were mounted in Elvanol between glass slides and coverslips and viewed vertically using a Zeiss Confocal Laser Scanning Microscope (Model LSM 710 NLO).

### *In situ* hybridization

*In situ* hybridizations of paraffin sections with DIG labeled probes were performed as described in (Schmidl et al., 2006). *Cre* and *YFP* mRNA specific RNA probes were generated by PCR using cDNA from cartilage of *Col10CreYFP^+^* mice as template. (All primer sequences see supplementary material Table S1.)

### Cell culture

For isolation of CDOP cells, growth plates were prepared from P6–P7 wild type and *Col10CreYFP^+^* long bones as described above and subjected to 3 subsequent steps of enzymatic digestion at 37°C: fraction 1 was collected after 15 min digestion with 0.1% trypsin in PBS; fraction 2 and fraction 3 were recovered after additional treatments with 0.1% collagenase P for 15 and 30 min, respectively, in DMEM/Ham's F12 containing P/S and 10% fetal calf serum. The residual cartilage was dissociated by pipetting and contained more than 99% proliferating and hypertrophic chondrocytes (fraction 4). Cells of fraction 1 (total ∼ 0.5–1×10^4^ cells from five *Col10CreYFP*^+^ pups from the same litter) and fraction 2 (2–4×10^4^ cells) were plated at 3–5×10^3^ cells/well (F1) or 2–4×10^4^ cells/well (F2) on fibronectin coated 12-well dishes for cell sorting of YFP^+^ cells. For immunofluorescence they were plated in chamber slides (10^3^ cells/well) in stem cell medium (αMEM containing 10% selected FCS, 1000 U/ml LIF (Millipore), 10 ng/ml EGF, 10 ng/ml PDGF-BB (R&D Systems), 100 U/ml penicillin/streptomycin (P/S) for 7–12 days and analyzed by immunofluorescence. For osteogenic differentiation, YFP^+^ sorted F2 cells were expanded for 2 weeks in α-MEM medium containing 10% FCS, P/S, 10 mM β-glycerophosphate and 50 µg/ml Na-ascorbate.

Trabecular osteoblasts were prepared by dissecting entire growth plates including adhering spongiosa from P5 *Col10CreYFP^+^* tibiae and femora; bony trabeculae were carefully separated from the cartilaginous growth plates under the binocular and subjected to digestion with 0.1% collagenase, 0.1% trypsin as above for 45 min at 37°C under gently shaking.

For the isolation of YFP-labelled endosteal cells, long bones (femora, tibia and humeri) from P20 *Col10CreYFP^+^* mice were carefully cleaned from adhering periosteum and soft connective tissue. After removing the epiphyses, bone marrow cells were removed by flushing the bone shafts with DMEM/Ham's F12 medium containing P/S. The remaining bone shafts were crushed and digested three times for 30 min with 1 mg/ml bacterial collagenase P (Roche) in DMEM/Ham's F12 medium. Collagenase-released cells were combined and used for FACS analysis (see below) or cultured on fibronectin-coated dishes for 1 week in DMEM/Ham's F12 containing 5% FCS and P/S for immunofluorescence.

Mouse MC3T3-E1 cells were cultured in αMEM containing 10% FCS, P/S, 10 mM β-glycerophosphate and 50 µg/ml Na-ascorbate.

### Analytical and preparative FACS

For preparative cell sorting, cells were washed once with PBS and detached with a trypsin/EDTA solution (0.1% trypsin, 0.25% EDTA in PBS) at 37°C for 5 min. Cells were centrifuged at 1200 rpm for 5 min, washed again with PBS and re-suspended in sorting buffer (PBS, 2% BSA, 5 mM EDTA). Before cell sorting, the cell suspension was filtered with a cell strainer and the cells were kept on ice. YFP-positive and -negative cells were sorted into 15 ml plastic tubes containing αMEM modification medium (10% FCS, 2× P/S, 1× Fungizone) with the MoFlo Legacy cell sorter (DakoCytomation) (kindly performed by Uwe Appelt).

For analytical FACS analysis, endosteal cells freshly isolated from P20 *Col10CreYFP^+^* long bones as described above were fixed in 4% paraformaldehyde, permeabilized with 0.1% Triton X-100 and stained with Goat-FITC-anti GFP (Abcam #6662), anti CD45 (Merck-Millipore #04-1102), rabbit anti Osx (Abcam #22552), Goat anti Ocn (LifeSpan BioSciences LS-C$2094) and analyzed using a Beckton-Dickinson Calibur.

### RT-PCR

RNA was extracted from unfixed, sorted YFP-positive and -negative CDOPs, trabecular osteoblasts or endosteal cells with RLT buffer using the Qiagen RNeasy kit and cDNA was prepared from RNA by reverse transcription (In VitroGen). CDNA was amplified with Taq polymerase (Qiagen) and gene specific primer pairs (see below) in a 35-cycle PCR. For semiquantitative expression analyses, starting cDNA amounts were adjusted to cyclophilin a in a 22-cycle PCR. All primer pairs annealed at 60°C except for *Col1a1* primers which annealed at 57°C. RT-PCR was performed as described in (Surmann-Schmitt et al., 2009). Quantitative Real time PCR was performed on a BioRAD CFX96 cycler as described in Hattori et al. (Hattori et al., 2010). (Primer sequences are listed in supplementary material Table S1.)

## RESULTS

### Identification of a chondrocyte-derived progeny of osteoblasts in the spongiosa of reporter mice

**Fig. 1.**
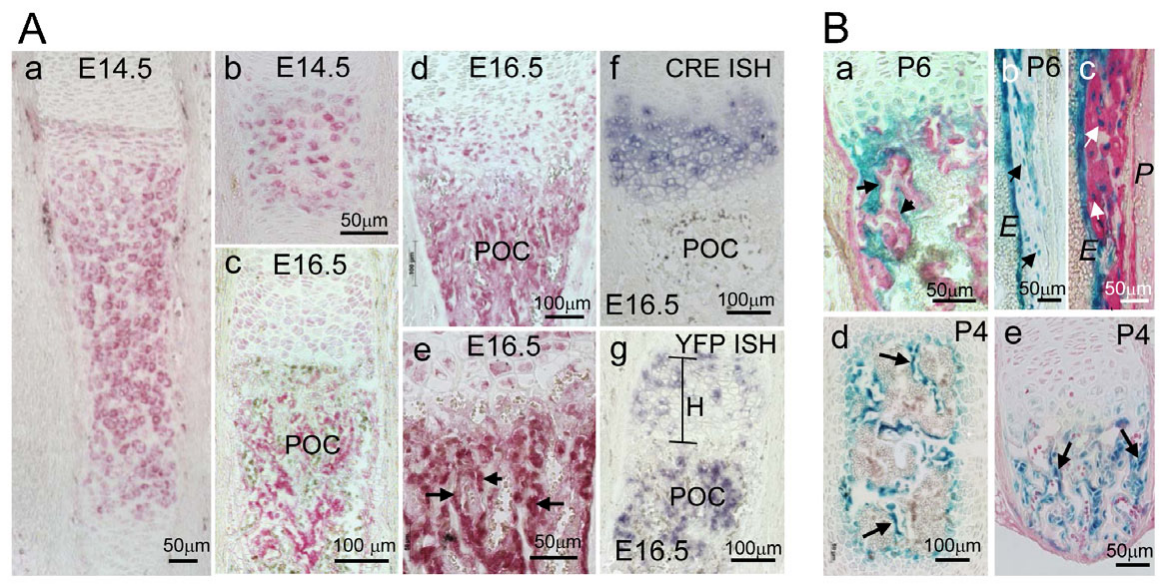
Lineage tracing reveals a progeny trabecular osteoblasts derived from Col10a1 expressing cells. (A) Anti YFP staining of *Col10Cre;YFP^+^* bones at E14.5, before bone marrow formation, is restricted to hypertrophic chondrocytes; (a) tibia, (b) digit; (c–e). At E16.5 YFP^+^ cells are visible in the spongiosa; (c) E16.5 ulna, (d,e) E16.5 tibia. (f) *In situ* hybridization shows that Cre is expressed exclusively in hypertrophic chondrocytes (see also supplementary material Fig. S1). (g) YFP is expressed both in hypertrophic chondrocytes, ‘H’, and in cells of the spongiosa; (f,g) E16.5 humerus. POC = Primary ossification center. (B) X-gal stained sections of *Col10Cre;R26R* mice (a) P6 radius; (b,c) P6 humerus; (d) P4 vertebra and (e) P4 rib reveals β-gal activity in osteoblast-like cells (blue) lining trabecular bone spicules (arrows in a,d,e), in endosteal cells (*E* in b,c), and in osteocytes (arrows in b,c). (c) Cells in the periosteum (*P*) do not stain for β-gal. Counterstaining of the extracellular matrix with anti Col1 (red).

In order to follow the cell fate of hypertrophic growth plate chondrocytes during endochondral ossification, *BACCol10Cre* deleter mice were mated to *ROSA26LacZ (R26R)* and *ROSA26YFP* mice, and the reporter gene activity in the offspring was followed during fetal and postnatal development by histochemistry and *in situ* hybridization. In the tibia of *BACCol10Cre;ROSA26YFP* (hereafter referred to as *Col10CreYFP^+^*) embryos at E14.5, before onset of bone marrow formation, YFP was detected by immunostaining exclusively in hypertrophic chondrocytes (Fig. 1Aa,b). After E16.5, following cartilage resorption and bone marrow formation, unexpectedly substantial YFP expression was in addition observed in the primary ossification center (POC) of *Col10CreYFP^+^* long bones both by immunostaining (Fig. 1Ac–e) and by *in situ* hybridization (Fig. 1Ag). In contrast, *Cre* mRNA was only detected in hypertrophic chondrocytes, but not in the spongiosa, periosteum or cortical bone by *in situ* hybridization, thus confirming the specificity of *Cre* expression under the *BACCol10a1* promoter for hypertrophic chondrocytes only (Fig. 1Af; supplementary material Fig. S1; see also Golovchenko et al., 2013).

**Fig. 2.**
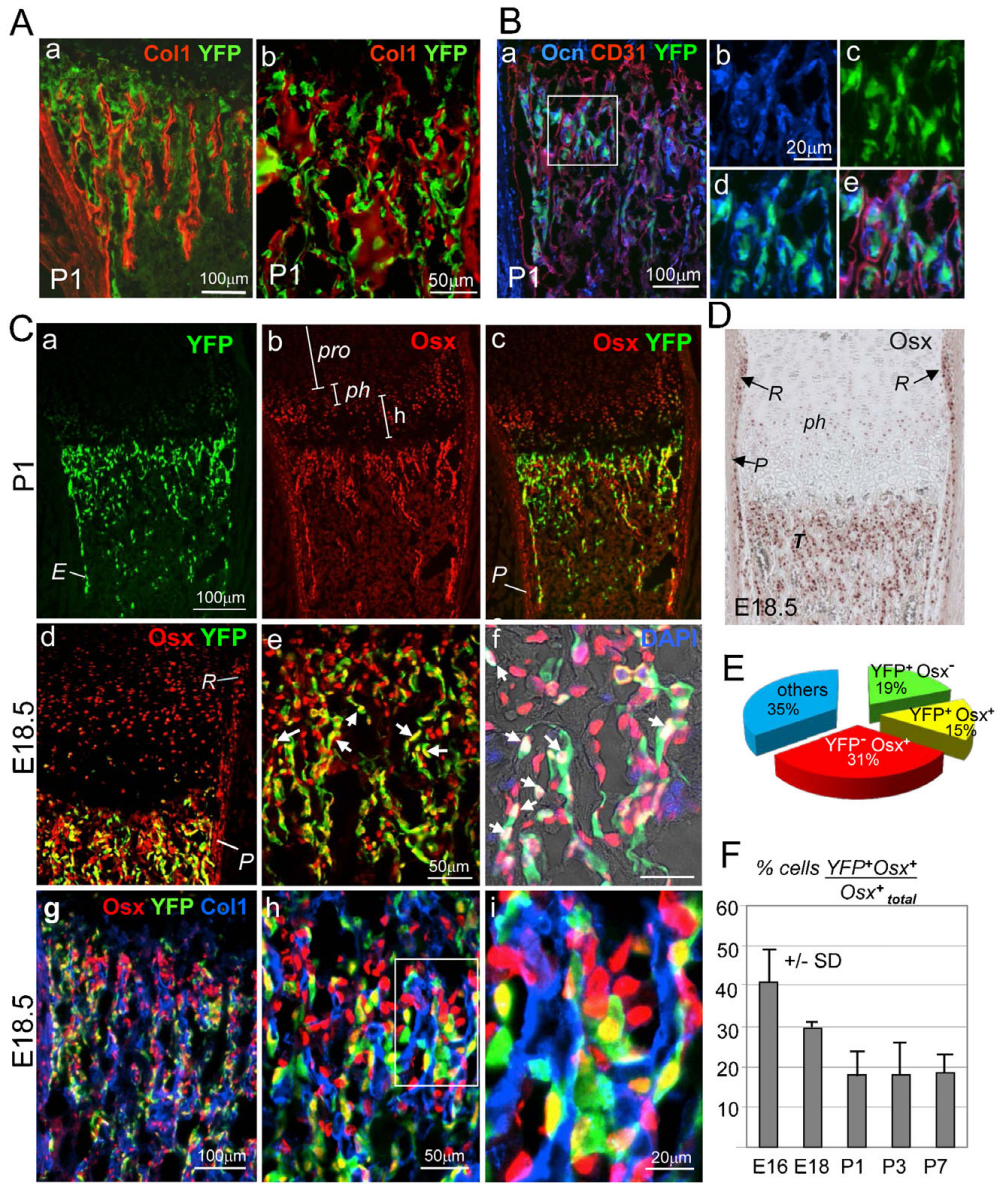
Dual origin of trabecular osteoblasts in the spongiosa of Col10CreYFP^+^ specimens. (A) Co-immunostaining for YFP and Col1 of a P1 *Col10CreYFP^+^* tibia shows that YFP^+^ cells in the spongiosa are to a large part associated with Col1^+^ trabeculae. Panels a and b show different magnifications. (B) Triple staining of a *Col10CreYFP*^+^ tibia (P1) for YFP (green), osteocalcin (Ocn, blue and CD31 (red), confirms the presence of YFP^+^Ocn^+^ osteoblasts in the spongiosa, whereas endothelial cells are YFP negative. (b–e) Enlarged images of the boxed area in panel a showing single channels (b,c), two channels (d) and all three channels (e). (Ca–f) Immunofluorescence staining for Osx (red) and YFP (green) shows that a substantial number of Osx^+^ cells in the spongiosa and endosteum ‘*E*’ of P1 (a–c) and E18.5 (d–i) tibiae are YFP^+^ (yellow cells in c–e; white cells in f). In the periosteum ‘*P*’ in, all Osx^+^ cells are negative for YFP (c,d). *pro* = proliferating zone, *ph* = prehypertrophic zone; *h* = hypertrophic zone. (g–i) Triple immunostaining for Osx, Col1 and YFP: most YFP^+^Osx^+^ cells (yellow) and YFP^−^Osx^+^ cells (red) are associated with trabeculae, stained for Col1 (blue). (D) Immunostaining of paraffin sections shows Osx^+^ cells in the groove of Ranvier ‘*R*’, periosteum ‘*P*’, pre-hypertrophic chondrocytes ‘*ph*’ and trabecular bone ‘*T*’. E18.5 *Col10CreYFP^+^* tibia. (E) Pie chart showing the relative amounts of chondrocyte-derived (YFP^+^) cells of all DAPI^+^ cells and other cells in the spongiosa of P2 tibiae. 15% of all DAPI^+^ cells are YFP^+^Osx^+^; 19% are YFP^+^Osx^−^ cells and thus not osteogenic; 31% are Osx^+^YFP^−^. Cells were counted in the trabecular zone in five humeri or tibia sections (100–300 cells per field). (F) Bar graph showing ratios of YFP^+^Osx^+^ double positive (yellow) cells to total Osx^+^ cells (red and yellow) in the spongiosa at various stages of development. Error bars: SD.

YFP expressing cells were primarily associated with subchondral trabecular bone and the endosteum (arrows in Fig. 1Ae; see also Fig. 2A), and therefore represented most likely osteoblasts. β-galactosidase positive (β-gal^+^) cells of epithelial, osteoblast like morphology lining trabecular and endosteal bone were observed in sections of *Col10CreR26R* long bones, ribs, and vertebrae (Fig. 1Ba,d,e, arrows). Importantly, neither YFP^+^ nor β-gal^+^ cells were ever detected in the periosteum (Fig. 1Bc and Fig. 2Cc,d).

Immunofluorescent co-staining for YFP and type I collagen (Col1) on frozen sections of long bones of *Col10CreYFP****^+^*** mice revealed that most YFP^+^ cells were aligned along Col1^+^ bone trabeculae in the primary ossification center (POC) (Fig. 2Aa,b and Fig. 2Cg–i). Some YFP^+^ cells in the spongiosa co-stained also for Ocn, a marker for mature osteoblasts (Fig. 2B), indicating that cells of chondrogenic origin have further differentiated along the osteogenic lineage. Co-staining for the endothelial marker CD31 (PECAM), however, revealed no double positive cells in the spongiosa of P2 *Col10CreYFP****^+^*** mice (Fig. 2Be).

Thus, these findings strongly indicate the existence of a progeny of osteoblasts in the spongiosa that must have originated from *Col10a1* expressing chondrocytes in the growth plate. This is consistent with the results of three recent cell tracking studies using non-inducible and tamoxifen-inducible *Col10Cre* (Yang et al., 2014a; Yang et al., 2014b), *Col2a1Cre* (Yang et al., 2014a) or *OsxCre* and *AgcnCre* (Zhou et al., 2014) lineage tracing systems to follow the cell fate of hypertrophic chondrocytes in the fetal and postnatal mouse growth plate.

### A high percentage of chondrocyte derived, YFP^+^ cells in the primary spongiosa of embryonic long bones are osteogenic

**Fig. 4.**
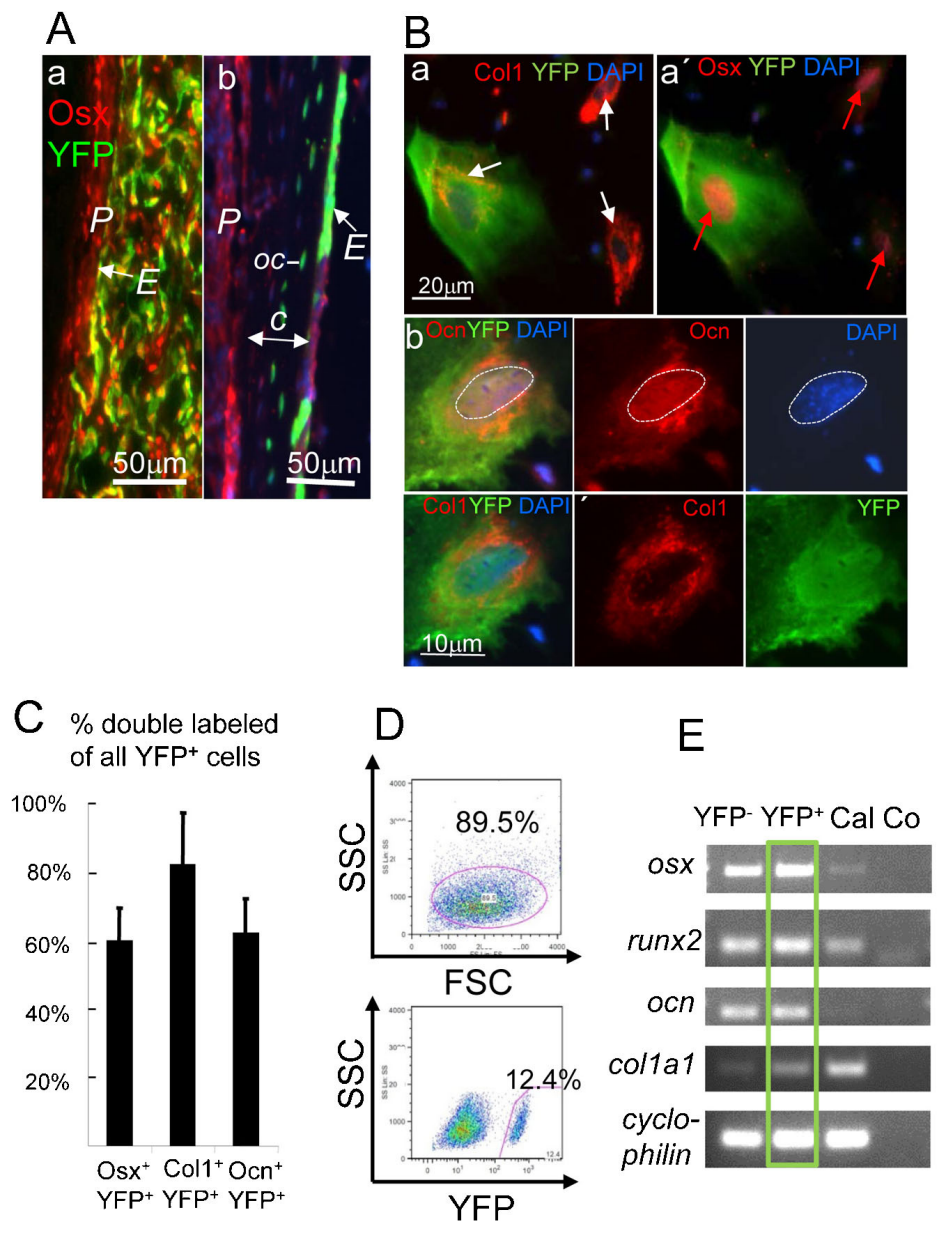
Expression of osteogenic genes in cultured endosteal cells. (A) Chondrocyte-derived YFP^+^Osx^+^ endosteal cells adjacent to the periosteum ‘*P*’ of a P2 tibia (a) and the cortical bone ‘*c*’ in P5 tibia (b) of *Col10CreYFP^+^* mice. Note in (b) YFP^+^ osteocytes (*oc*) are visible in the cortical bone. *E* = Endosteum. (B) Immunofluorescence triple staining of endosteal cells isolated from long bones of 3-week-old *Col10Cre;YFP^+^* mice for osteoblast-specific proteins after 1 week of culture. YFP^+^ cells co-stained for Col1 (white arrow) and nuclear Osx (red arrows, a,a′), and Col1 and Ocn (b). Note: anti Osx and anti Ocn were counterstained with Cy5-anti Rabbit IgG, but depicted in red. All panels in b show the same cell. (C) About 60% of all YFP^+^ cells are also positive for Osx and Ocn, and about 80% for YFP and Col1; four fields of 200–300 cultured cells each were counted. Error bars: SD. (D) FACS blot showing separation of YFP^+^ and YFP^−^ endosteal cells after 1 week in culture; upper panel: gating for viable cells (89.5% of total cells). Lower panel: gating for YFP^+^ cells (12.4%). (E) RT-PCR analysis of RNA isolated from sorted YFP^+^ and YFP^−^ endosteal cells as in (D). YFP^+^ cells (green box) express all four osteoblast marker genes *osx* (*Sp7*), *runx2* (*cbfa-1*), *ocn* ( = *bglap*, *osteocalcin*), and *col1a1*; Cal = calvarial cDNA; Co = water control.

Immunofluorescent double staining of P1 tibiae from *Col10CreYFP****^+^*** mice for YFP and Osx revealed that a substantial proportion of Osx^+^ cells in the primary spongiosa and the endosteum (“*E”* in Fig. 2Ca and Fig. 4A) were also YFP^+^ (yellow cells in Fig. 2Cc–e), indicating that they were derived from Col10a1 expressing cells. Similar patterns were seen in frozen sections of humeri, tibiae, and femora from E16.5-P10 *Col10CreYFP****^+^*** bones. Importantly, in the periosteum (“*P*” in Fig. 2Cc,d), all Osx^+^ cells were negative for YFP, also in the groove of Ranvier (“*R*” in Fig. 2Cd,D), which has been identified as the origin of periosteal osteoblasts (Langenskiöld, 1998; Shapiro et al., 1977).

For quantitative assessment of the contribution of YFP^+^ cells to bone forming cells in the spongiosa, sections of E16.5 to P7 epiphyses were triple stained for DAPI, YFP and Osx, and the labeled cells were counted in the spongiosa and endosteum of five different specimens. This revealed that at P2 15% of all DAPI^+^ bone marrow cells were YFP^+^Osx^+^ double positive (yellow cells in Fig. 2Cc–e,g–I; white cells in Fig. 2Cf) and 19% were YFP^+^Osx^−^ (Fig. 2E). In total 34% of all bone marrow cells were YFP^+^, indicating that a substantial fraction of chondrocyte-derived YFP^+^ cells in the spongiosa represented either uncommitted osteoprogenitor cells or bone marrow cells with other cell fates. In the spongiosa at E18.5, about 2/3 of the Osx^+^ cells were YFP negative (Fig. 2Cd–i,E). This population of Osx^+^ YFP^−^ cells most likely represents the progeny of periosteum-derived osteoprogenitor cells invading the bone marrow along with endothelial cells (Maes et al., 2010; Ono et al., 2014). Triple staining of E18.5 *Col10CreYFP^+^* bones for Col1, YFP and Osx confirmed that both chondrocyte-derived Osx^+^ cells (yellow) as well as periosteum derived Osx^+^ cells (red) co-aligned along the same trabeculae (blue for Col1) and did not separate into distinct zones (Fig. 2Cg–i).

When analyzing the percentage of YFP^+^Osx^+^ double positive cells (yellow) within the Osx^+^ population, it turned out that at E18.5 about 30% of all Osx^+^ cells were also YFP^+^ (Fig. 2F). Interestingly, the percentage of YFP^+^Osx^+^ cells was higher at E16.5 with 40%, but decreased to less than 20% at postnatal day P7 (Fig. 2F).

**Fig. 3.**
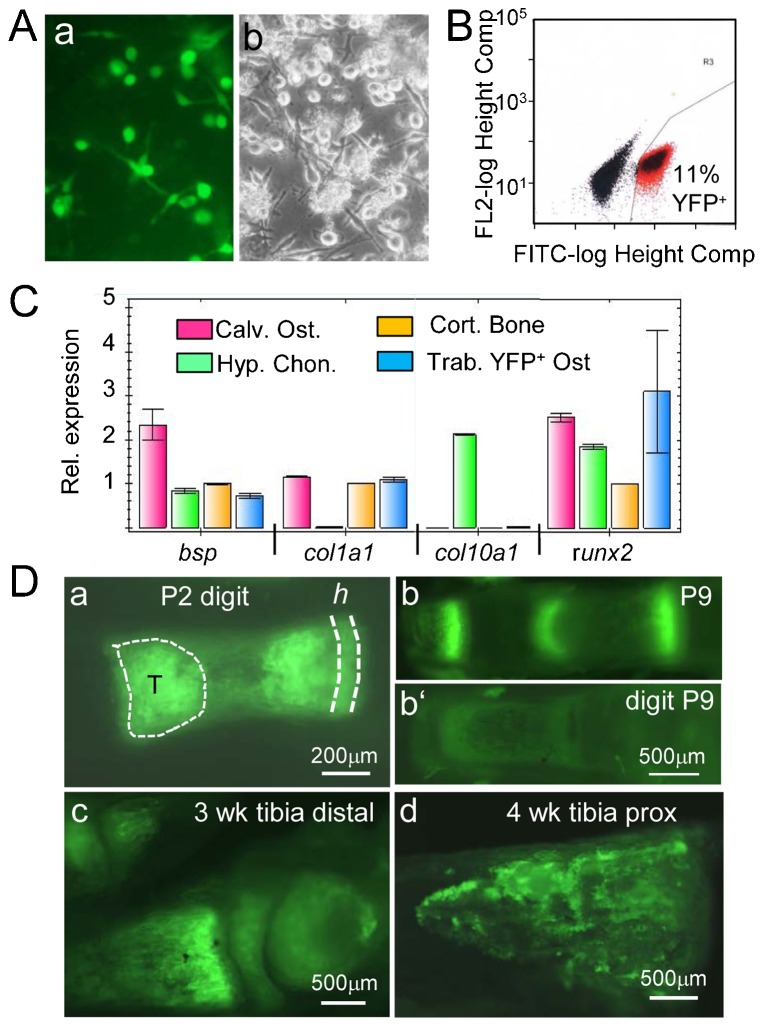
YFP^+^ labeled trabecular cells express bone markers. (A) Culture of trabecular cells isolated by microdissection and collagenase digestion from the trabecular zone (“T” in Fig. 3Da) of P7 *Col10CreYFP^+^* tibiae and femora; (a) endogenous YFP fluorescence; (b) phase contrast. (B) 11% of cultured trabecular cells are YFP^+^. (C) Bar graph showing qRT-PCR results for osteogenic genes, *Bsp*, *Col1a1*, *Col10a1* and *Runx2* using RNA from sorted YFP^+^ trabecular cells (Trab. YFP^+^ Ost.), cultured calvarial osteoblasts (Calv. Ost.), hypertrophic chondrocytes (Hyp. Chon.), as well as RNA from cortical bone tissue (Cort. Bone) as a positive control. Error bars; SD. (D) Endogenous YFP fluorescence in hypertrophic cartilage ‘*h*’, bone marrow and spongiosa in several postnatal *Col10CreYFP^+^* bones. Depicted are freshly dissected unfixed skeletal elements, left intact (a,b) or split sagitally in two halves (c,d). (a) P2 digit; T = trabecular zone; (b) P9 digit, (b′) comparable region of Cre negative digit; (c) distal region of a tibia at 3 weeks; (d) proximal region of a tibia at 4 weeks.

In order to confirm the expression of osteogenic genes by YFP^+^ labeled spongiosa cells, growth plate associated bone trabeculae were isolated from the spongiosa of femoral heads and tibia plateaus of P5 *Col10CreYFP^+^* mice by microdissection. After digestion of trabeculae with trypsin/collagenase, released cells were cultured for 7 days (Fig. 3A), dissociated, and YFP^+^ cells were isolated by fluorescence activated cell sorting (FACS) (Fig. 3B). FACS sorted cells were cultured for one more week and then subjected to QRT-PCR analysis. This revealed significant expression of osteogenic markers such as *Bsp*, *Col1a1*, and *Runx2* at levels comparable to calvarial osteoblasts and cortical bone (Fig. 3C).

### Contribution of chondrocyte-derived YFP^+^ cells to endosteal and cortical bone

The results of antibody staining for Osx and YFP (Fig. 2) indicated a substantial contribution of chondrocyte-derived cells to trabecular, endosteal, and cortical bone formation. This notion was confirmed by YFP fluorescence analysis in postnatal bones of *Col10creYFP^+^* mice. Strong YFP fluorescence was seen in hypertrophic cartilage, trabecular bone and endosteum of freshly isolated, unfixed long bones, as shown for digits, metatarsals and tibiae (Fig. 3D).

For quantitative assessment of the contribution of YFP**^+^** labeled cells to total osteogenic cells in the endosteum (*E*, as depicted in Fig. 4A), endosteal cells were harvested from the bone shafts of P20 *Col10CreYFP^+^* tibiae and femora by collagenase digestion and stained with antibodies for YFP, CD45, Osx, and Ocn. Flow cytometry analysis revealed that 36% of all non-immune (CD45^−^) cells were YFP^+^ (supplementary material Fig. S2). Co-staining with anti YFP and anti Osx or anti Ocn showed that 54% of all endosteal Osx**^+^** cells and 25% of all Ocn**^+^** cells in the CD45^−^ population, respectively, were YFP**^+^**. The two size populations visible in supplementary material Fig. S2D,E probably reflect smaller osteoprogenitor cells and mature osteoblasts. Gating for YFP**^+^** cells showed that 88% all YFP**^+^** endosteal cells were Osx^+^, and 18% were Ocn^+^ (supplementary material Fig. S2). This confirms that the Col10Cre derived YFP^+^ cells contribute substantially to endosteal bone.

To confirm the osteogenic nature of Col10CreYFP^+^ labeled endosteal cells *in vitro*, endosteal cells were isolated by collagenase digestion of 3 weeks *Col10CreYFP^+^* bone shafts, cultured for 1 week and triple stained with antibodies against Col1, Osx and Ocn (Fig. 4B). Numerous YFP^+^ as well as YFP^−^ cells co-stained for Col1/Osx, and for Col1/Ocn (Fig. 4Ba–c). Notably, the secreted protein Ocn and the extracellular matrix protein Col1 appeared partially in different cytoplasmic compartments (Fig. 4Bb). Quantitative analysis of fluorescent cells (counting 200–300 cells in four culture dishes) revealed that about 60% of all YFP^+^ cells were also positive for Osx or Ocn, and about 80% positive for YFP and Col1 (Fig. 4C).

Next we analyzed expression of bone typical genes in YFP^+^ cells from 8 d endosteal cell cultures after separation from YFP^−^ cells by FACS (Fig. 4D). RT-PCR analysis of RNA isolated from both YFP^+^ (12% of all cells) and YFP^−^ cells revealed the expression of four major osteogenic genes, *osx*, *runx2*, *ocn (Bglap)*, and *col1a1* (Fig. 4E).

The contribution of chondrocyte-derived osteoblasts to cortical bone formation was determined in paraffin sections of P17 and P35 tibiae and femora of *Col10Cre;R26R* mice after staining with X-gal and nuclear fast red. Cell counting of five sections each showed that about 40% +/− 16.5% of all osteocytes in the cortical bone matrix of P17 tibiae were β-gal^+^, the majority of them being located at the endosteal side of the cortical bone shaft (supplementary material Fig. S3A). Similar, about 50% of osteocytes in the cortex of P35 tibiae were β-gal^+^ (supplementary material Fig. S3B).

### Identification of a novel, small chondrocyte–derived osteoprogenitor cell type (CDOP) in the lower hypertrophic zone of the growth plate

While the above findings as well as similar findings by others (Yang et al., 2014a; Yang et al., 2014b; Zhou et al., 2014) provide strong evidence for transdifferentiation of hypertrophic chondrocytes to osteoblasts, the question remained how small osteoprogenitor cells can emerge for the substantially larger hypertrophic chondrocytes with diameters of 15–20 µm. In attempts to identify the origin of the Col10Cre-induced YFP labeled osteoprogenitor cells in the hypertrophic region, we analyzed growth plates isolated by microdissection of P5–P7 ColCreYFP^+^ tibiae, femora, and humeri (Fig. 5A) by confocal microscopy. Detailed analysis of the chondro-osseous junction at serial z-axis levels revealed the presence of small YFP^+^Col1^+^ cells of 4–6 µm diameter in the lowest zone of hypertrophic chondrocytes adjacent to the cartilage-bone interface (Fig. 5B,C). Analysis of sagittal vibratome sections of P5 epiphyses including the growth plate and chondro-osseous junction confirmed the presence of small YFP^+^ cells in the lower hypertrophic zone (Fig. 5D).

The analysis of Z-stacks of 24 layers in 1 µm distance (supplementary material Fig. S4) by confocal microscopy confirmed that the small Col1^+^YFP^+^ cells appeared in the bottom layer of hypertrophic chondrocytes, approximately 25 µm adjacent to cartilage-spongiosa interface (Fig. 5C). The number of Col1^+^YFP^+^ cells increased towards the spongiosa (Fig. 5E), indicating proliferation and increasing differentiation to osteoblasts. Similar, confocal microscopy of growth plates immunostained for Osx and YFP showed small double positive cells in the same zone close to the cartilage-spongiosa interface (Fig. 5F).

**Fig. 6.**
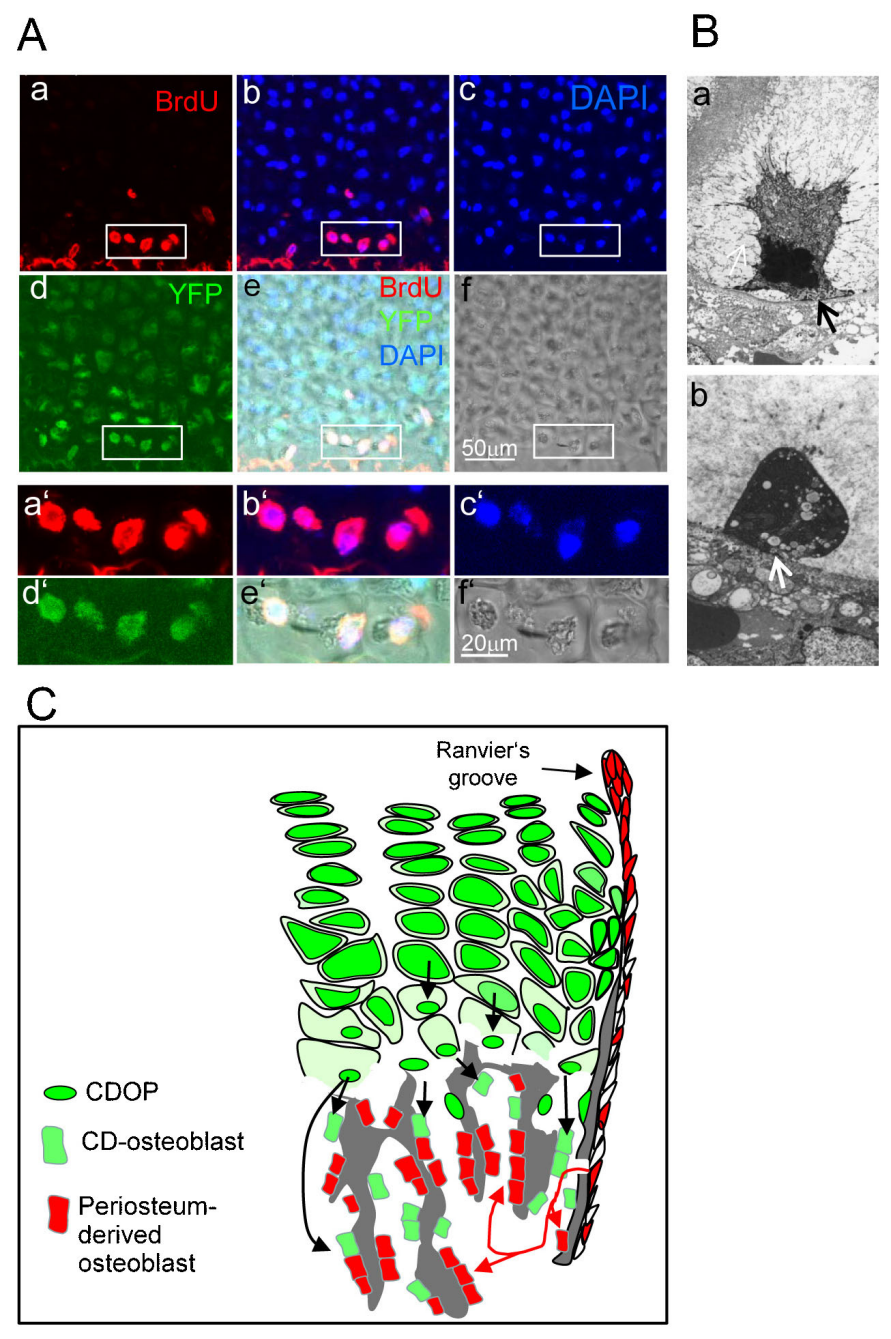
YFP^+^ CDOP cells in the chondro-osseous junction are mitotically active. (A) BrdU was injected peritoneally into female Col10CreYFP+ mice 12 h before delivery, and mitotically active cells in the growth plates of the newborn CreYFP^+^ pups were analyzed by confocal microscopy after co-staining of 25-µm sagittal vibratome sections of tibia- and femur epiphyses with anti BrdU (a,b,e), DAPI (b,c,e) anti YFP (d,e). YFP^+^BrdU^+^ CDOP cells (white rectangles) in the lowest zone of hypertrophic cartilage lacunae. a′–f′ higher magnifications of the regions marked by white rectangles in a–f. (B) Electronmicrographic images of condensed terminal hypertrophic chondrocytes showing a condensed nucleus and extensive cytoplasmic vacuolization. The plasma membrane has withdrawn from its attachment to the pericellular matrix, but it maintains an asymmetric attachment to the terminal transverse septum (arrows). (a) Shrunken cell with remaining cell protrusions and contacts to the extracellular matrix. (b) Shrunken cell with protrusions retracted. Fixation with Ruthenium hexamine trichloride (Farnum and Wilsman, 1987). Magnification ×5000 (images kindly provided by Dr. C. E. Farnum, Cornell University, Ithaca, NY). (C) Model depicting dual origin of trabecular osteoblasts. A substantial proportion of trabecular osteoblasts in the spongiosa and endosteum (green cells) originate from Col10Cre expressing hypertrophic chondrocytes that give rise to chondrocyte-derived osteoprogenitor cells (CDOPs), which are small, proliferating cells that express osteogenic markers. The majority of trabecular osteoblasts are derived from Osx^+^ osteoprogenitor cells invading together with endothelial cells from the periosteum (red cells) (Maes et al., 2010; Ono et al., 2014).

In order to show whether these cells which we named CDOP (Chondrocyte-Derived OsteoProgenitor) cells are mitotically active, BrdU was injected intraperitoneally into pregnant *Col0Cre;YFP^+^* mice one day before delivery. Vibratome sections (25 µm) of the growth plates of the newborn pups were stained with antibodies against BrdU and YFP and analyzed by confocal microscopy. Strong BrdU uptake was seen in small, YFP^+^ CDOP like cells in the lowest zone of hypertrophic chondrocytes within the growth plate (Fig. 6A). Size and position of these cells resembled very much the “condensed hypertrophic chondrocytes” described ultrastructurally by Farnum and Wilsman (Farnum and Wilsman, 1987) in the lowest row of closed lacunae of hypertrophic growth plate cartilage which make a direct asymmetrical attachment of the plasma membrane with the last transverse septum (Fig. 6Ba,b, arrows). These cells have a condensed nucleus and extensive cytoplasmic vacuolization and fill only a small part of the space of its lacuna. Initial attachments to the pericellular and territorial matrices (Fig. 6Ba) eventually disappear, except at the last transverse septum (Fig. 6Bb) (Farnum and Wilsman, 1987). Whether these actually represent the described CDOP cells, however, remains to be confirmed.

**Fig. 7.**
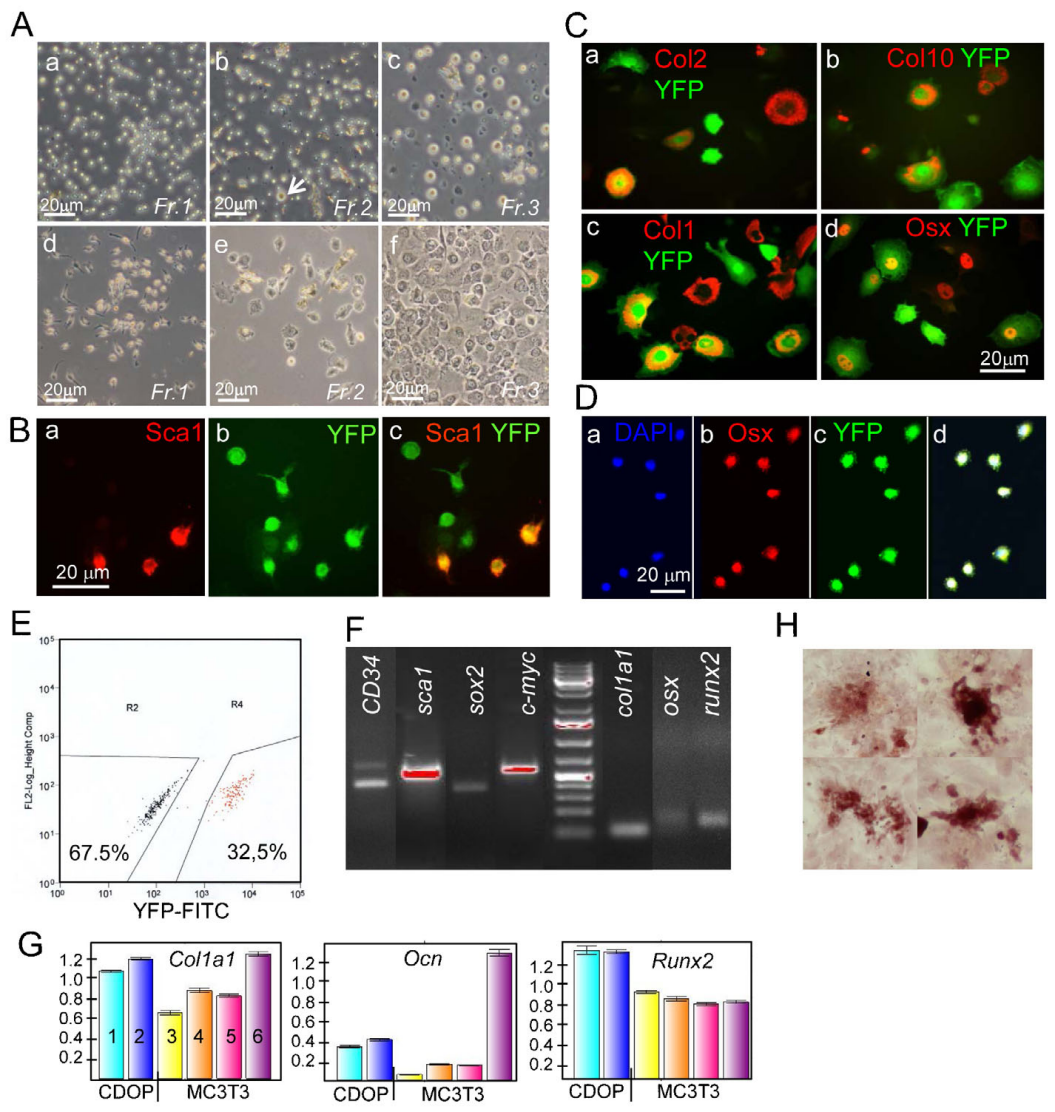
Isolation of YFP^+^ CDOP cells from Col10CreYFP^+^ growth plates. (A) Isolation of different cell populations from growth plates by fractionated trypsin-collagenase digestion. (a,d) fraction 1 (15 min trypsin); (b,e) fraction 2 (+15 min collagenase), arrow: chondrocyte; (c,f) fraction 3 (+30 min collagenase). (a–c) cells after 24 h in culture, (d–f) after 11 (d) or 7 days (e,f) in culture. Note: fraction 3 contains mostly chondrocytes. Fr, fraction. Fluorescence analysis of fraction 1 cells after 24 h in culture revealed that about 25% of all cells showed YFP fluorescence (see supplementary material Fig. S5). (B) Immunofluorescence analysis of 11 day fraction 2 cell cultures shows numerous small, YFP^+^ cells (∼5 µm diameter) co-expressing the stem cell marker Sca1 (a–c). (C) Immunofluorescence analysis of fraction 3 11-day cell cultures shows large YFP^+^ cells expressing chondrogenic markers Col2 (a) and Col10 (b) as expected, but also YFP^+^ cells expressing Col1 (c), and Osx (d) which may be dedifferentiating cells or cells undergoing osteogenic transformation. (D) YFP^+^ fraction 2 cells sorted as in E were cultured for another week in chamber slides and stained with anti Osx. (E) Sorting of fraction 2 cells by FACS after expansion for 10 days rendered about one third YFP^+^ cells. (F) RT-PCR analysis of YFP^+^ sorted fraction 2 cells as in E shows the expression of stem cell genes (*CD34*, *sca1*, *sox2*, *c-myc*), and of genes of the osteogenic (*col1a1*, *osx*, *runx2*) lineage. (G) Quantitative RT-PCR analysis of YFP^+^ sorted fraction 2 cells as above, but cultured for another 14 days in under osteogenic stimulation (β-glycerophosphate and Na-ascorbate). Expression levels of *Col1a1*, *Ocn* and *runx2* in two different culture dishes of CDOP derived cells (1,2) were compared to differentiating MC3T3 cells cultured 4 days without (3) or with osteogenic stimuli for 4, 7, and 14 days, respectively (4,5,6). (H) Alizarin red positive osteoblast nodules in YFP^+^ sorted fraction 2 cells cultured for 2 weeks.

Attempts were made to isolate and further characterize CDOPs from *Col10CreYFP^+^* growth plates *in vitro*. By sequential enzymatic digestion of isolated P5–P7 growth plates (as shown in Fig. 5A) with trypsin for 15 min, followed by 15 min and 30 min collagenase, three fractions were obtained which were cultured on fibronectin-coated coverslips or culture dishes. After 24 h fraction 1 consisted exclusively of small cells (∼4–5 µm) (Fig. 7Aa). Their size corresponded to the size of CDOPs observed by confocal microscopy. Cells in fraction 2 were of similar appearance, but contained few chondrocytes (Fig. 7Ab, arrow), while fraction 3 contained mostly proliferating and hypertrophic chondrocytes and some fibroblastic cells (Fig. 7Ac). When live cells were analyzed after 24 h for endogenous YFP fluorescence, about 20–25% of the cells in fraction 1 (supplementary material Fig. S5) and 30–35% of Fraction 2 cells (not shown) were positive. The bulk of YFP^−^ cells in fraction 1 and 2 did not attach to fibronectin-coated dishes and presumably represented bone marrow cells trapped in the residual trabecular fragments of the isolated growth plates.

After 7–12 days in monolayer culture, polygonal, fibroblastoid, and spindle shaped cells appeared in cultures of fraction 1 and 2 (Fig. 7Ad,e). Immunofluorescence analysis of fraction 2 revealed numerous small YFP^+^ cells staining for the stem cell marker Sca1 (Fig. 7Ba–c). Fraction 3 contained YFP^+^ chondrocytes and hypertrophic chondrocytes staining for Col2 and Col10 (Fig. 7Ca,b), as well as YFP^+^Col1^+^ and YFP^+^Osx^+^ cells, part of which may represent dedifferentiated chondrocytes or CDOP cells undergoing osteogenic transformation.

For further analysis of fraction 2 cells, cells were expanded for 10 days, and YFP^+^ and YFP^−^ cells were separated by FACS (Fig. 7E). After one additional week in culture cells almost all YFP^+^ cells co-stained positive for Osx (Fig. 7D). RT-PCR analysis of RNA isolated from YFP^+^ fraction 2 cells sorted as in Fig. 7E and analyzed without further culturing revealed expression of both stem-cell typical genes such as *CD34*, *sca1*, *sox2* and *c-myc*, as well as genes of the osteogenic lineage including *col1a1*, *osx*, and *runx2* (Fig. 7F). After additional 14 days in culture, we analyzed expression of osteogenic genes in sorted YFP^+^ cells by quantitative RT PCR. MRNA levels of *Col1a1*, *Ocn* and *Runx2* in YFP^+^CDOP cells were higher than those of differentiating MC3T3 osteoblasts cultured for 4–7 days (Fig. 7G). Only at d14 Col1a1 and Ocn expression was higher in MC3T3 cells than in CDOP fraction 2 cells which may be due to inadequate conditions for osteogenic differentiation of CDOP cells. Furthermore, the sorted and cultured YFP^+^ cells formed alizarin-red nodules, indicating bone matrix deposition, after two weeks of culture (Fig. 7H).

Altogether, these findings confirm that the population of small, 4–6 µm cells in fraction 2 isolated from the growth plates contained chondrocyte-derived cells with osteoprogenitor character. They represent most likely the CDOP cells observed by confocal microscopy in the chondro-osseous junction of the growth plate.

Nevertheless, the question remained how mature hypertrophic chondrocytes of 10–20 µm diameter in the fully hydrated form shrink to the “condensed” form (Farnum and Wilsman, 1989) with a similar size as CDOPs (4–6 µm). Although currently still speculative, one likely mechanism would be autophagy, an evolutionary conserved intracellular degradation process in all eukaryotic cells that acts as a cell protective mechanism under stress conditions and thus may prevent apoptosis (Yang and Klionsky, 2010). By sequestering dysfunctional organelles, proteins and membranes into double-membrane vesicles called autophagosomes, which later fuse with lysosomes, cells generate energy and remain viable and complete their life cycle (Kimura et al., 2007; Srinivas et al., 2009b; Yang and Klionsky, 2009). We observed strong reactions in hypertrophic chondrocytes with antibodies to the autophagy markers beclin-1 and LC3B (supplementary material Fig. S6), which is consistent with previous reports (Bohensky et al., 2007; Srinivas et al., 2009a; Zhang et al., 2013).

## DISCUSSION

In this study we followed the cell fate of hypertrophic chondrocytes during cartilage-bone transition in the developing mouse skeleton *in situ* using BACCol10Cre-induced YFP or LacZ expression as specific lineage tracers. We show that Col10cre expressing chondrocytes give rise to a progeny of osteoprogenitor cells appearing in the lowest zone of hypertrophic cartilage close to the chondro-osseous junction, which differentiate into a second pool of osteoblasts and contribute substantially – together with periosteum-derived osteoblasts – to trabecular, endosteal and cortical bone formation.

This conclusion relies on the premise that the YFP and LacZ reporter genes are continuously expressed under the ROSA26 promoter in the progeny of hypertrophic or prehypertrophic chondrocytes, once the ROSA26^fl/fl^ locus is activated by the Cre recombinase expressed by our BACCol10Cre lines. Exclusive *Cre* expression in hypertrophic chondrocytes, but not in osteoblasts nor in other skeleton related cells, was demonstrated here and in other studies by *in situ* hybridization (Golovchenko et al., 2013; Zhou et al., 2014). Therefore, the finding of strong LacZ and YFP expression in trabecular, endosteal and cortical osteoblasts, but not in osteoblasts of membranous bone, strongly indicates that a significant proportion of trabecular osteoblasts of the spongiosa is derived from Col10Cre expressing hypertrophic chondrocytes (see scheme in Fig. 6C). Furthermore, our findings are in agreement with three recent lineage tracing studies (Yang et al., 2014a; Yang et al., 2014b; Zhou et al., 2014) using different tamoxifen-inducible Cre lines, Col10Cre, Col2Cre or Agcn-Cre, and Cre-mediated reporter systems.

The question whether hypertrophic chondrocytes may transform into trabecular osteoblasts in the embryonic and juvenile growth plate of long bones during endochondral ossification rather than being eliminated by apoptosis has been an issue of controversy during the past decades (reviewed in detail by Shapiro et al., 2005). A large body of literature exists presenting evidence for apoptotic changes in hypertrophic chondrocytes in the growth plates of the Yucatan swine (Farnum and Wilsman, 1987; Farnum and Wilsman, 1989), chick sternum (Gibson, 1998; Gibson et al., 1995), mouse long bones (Amling et al., 1997), rabbit (Aizawa et al., 1997), and pig (Zenmyo et al., 1996). On the other hand, various studies have shown that under certain experimental conditions hypertrophic chondrocytes can transform into bone forming cells (Moskalewski and Malejczyk, 1989; Weiss, 1986; Roach, 1992; Roach et al., 1995; Galotto et al., 1994; Gentili et al., 1993; Gerstenfeld and Shapiro, 1996). Roach and co-authors (Roach, 1992; Roach et al., 1995; Erenpreisa and Roach, 1996) have provided ultrastructural evidence for asymmetric division of hypertrophic chondrocytes in chick bones with one daughter cell undergoing apoptosis, and the other transforming into an osteoblast. This osteogenic transformation was, however, only seen after cutting through embryonic chick femora and culturing the fragments for one week (Roach et al., 1995). In several other cell- and organ culture systems conversion from a typical cartilage-specific (*Col2a1*, *Agcn*, *Col10a1*) towards an osteogenic gene expression profile, including collagen I, has been demonstrated *in vitro* (Galotto et al., 1994; Gentili et al., 1993; Kirsch et al., 1992; Riminucci et al., 1998; Weiss et al., 1986). However, the possibility of contaminations with stem cells differentiating into osteoblasts could never be entirely excluded in these *in vitro* experiments (Weiss et al., 1986). In addition, concerns were raised whether the conclusions regarding osteogenic transformation of hypertrophic chondrocytes obtained under experimental conditions were applicable to the *in vivo* process of cartilage-bone transition, although transdifferentiation or metaplasia of cells has been documented for several cell types (Li et al., 2006; Medici et al., 2010; Takimoto et al., 2012; Thesingh et al., 1991; for a review, see Tosh and Slack, 2002). Therefore it is conceivable that a substantial fraction of hypertrophic chondrocytes may undergo transformation to osteogenic cells, while others are eliminated by cell death. The relative proportion of dying versus transdifferentiating cells most likely depends on the species, the developmental stage, the bone type and the activity of the particular growth plate.

The high proportions of YFP^+^ cells found in the spongiosa of E16.5 and E18.5 mice indicate that in the early phases of endochondral ossification chondrocyte-derived osteoblasts contribute substantially to trabecular bone formation. Their contribution declines during postnatal development. Given that postnatally the steady state level of YFP^+^ trabecular osteoblasts at all stages examined (P1, P5, P7) is approximately 20%, and that the life span of a murine osteoblast is approximately 12 days (Weinstein et al., 1998), we estimate that postnatally the rate of newly emerging hypertrophic chondrocyte-derived osteoblasts is only about 10% or less, as half of the chondrocyte-derived osteoblasts should have died after 6–7 days. In the E18.5 embryo, however the contribution of chondrocyte derived osteoblasts is with around 30% much higher. In the postnatal endosteum, however, more than 50% of all Osx^+^ cells were YFP^+^, and cortical bone of 2- to 5-week-old *BACCol10Cre;R26R* mice contained about 40% β-gal^+^ osteocytes. This difference between trabecular osteoblasts and osteocytes can be explained by the longer half-life of osteocytes, which is estimated to be 25 years in human bone (Knothe Tate et al., 2004). In the mouse osteocytes probably live until the bone matrix they reside in is replaced (Jilka et al., 2007).

Our findings strongly indicate that endochondral osteoblasts originate from two pools: (i) from perichondrium-derived osteoprogenitor (Colnot et al., 2004; Kusumbe et al., 2014; Maes et al., 2010) and (ii) from hypertrophic chondrocytes transdifferentiating into osteoblasts. In essence, all osteoblasts are derived from a common Sox9 expressing chondro-osteoprogenitor cell (Akiyama et al., 2005). As shown recently using lineage-tracing approaches with *Col2a1Cre* or *Sox9Cre* and inducible derivatives thereof, these chondro-osteoprogenitors continue to reside in the mesenchyme surrounding the cartilage element and invade the bone marrow from the perichondrium (Ono et al., 2014). This is in agreement with earlier studies showing that perichondrium-derived osteoprogenitors migrate in along with capillaries and mature then into osteoblasts found on trabecular and endosteal bone (Colnot et al., 2004; Kusumbe et al., 2014; Maes et al., 2010). In contrast to that, chondrocyte-derived osteoblasts (CDOP cells), described here and reported by others, do not migrate through the perichondrium, but transdifferentiate from hypertrophic chondrocytes into osteoblasts and participate in trabecular and endosteal, but not periosteal bone formation (Yang et al., 2014a; Yang et al., 2014b; Zhou et al., 2014). The fact that CDOP cells and their descendants express the reporter gene activated by Col10Cre which is expressed only in prehypertrophic and hypertrophic chondrocytes excludes the possibility that the CDOP cells are derived from the perichondral population of osteoprogenitor cells.

Thus, two distinct pathways of osteoblast development exist in endochondral ossification. According to Ono and colleagues (Ono et al., 2014) the perichondrial-derived population of osteoblast precursors contributes to about 80% of all endochondral osteoblasts, which is nearly compatible with an estimated 10% contribution of hypertrophic chondrocyte-derived osteoblasts (see above).

In the previous reports (Yang et al., 2014a; Yang et al., 2014b; Zhou et al., 2014) the question has remained open exactly where and how conversion of chondrocytes into osteoblasts occurs. One possible explanation would be that a subpopulation of *Col10a1* expressing prehypertrophic or early hypertrophic chondrocytes divides asymmetrically, remains proliferative and does not undergo hypertrophic, but osteogenic differentiation instead once the cell reaches the chondro-osseous front where it is exposed to signals from the bone marrow. However, even after extensive survey of *Col10CreYFP^+^* labeled growth plates by confocal microscopy we failed to identify small YFP^+^ cells within the early and upper hypertrophic zone except for the lowest zone, the site of CDOP cell detection.

In light of previous ultrastructural studies by Farnum and Wilsman on morphological changes of hypertrophic chondrocytes in the growth plate (Farnum and Wilsman, 1987; Farnum and Wilsman, 1989), we favor the concept that CDOPs emerge directly from hypertrophic chondrocytes in the lowest hypertrophic zone. The small size and location of CDOPs appears strikingly similar to the condensed cells observed in the lowest intact lacunae of 24% of all hypertrophic chondrocyte columns in growth plates of rat and Yucatan swine after fixation with ruthenuim hexamine trichloride (Farnum and Wilsman, 1987; Farnum and Wilsman, 1989). These condensed hypertrophic chondrocytes make only one asymmetrical attachment to the lowest transverse septum of the calcified cartilage and occupy only a fraction of the entire lacuna. The fact that each condensed chondrocyte is located in the lowest intact lacunae of a column of hypertrophic chondrocytes indicates that it is directly derived from the adjacent larger hypertrophic chondrocytes in the same column (Farnum and Wilsman, 1987; Farnum and Wilsman, 1989). Although the authors favored cell death by apoptosis as the likely cell fate of the condensed cells, they also included the possibility for alternate post-hypertrophic fates of these cells in bone marrow and endochondral ossification (Farnum and Wilsman, 1987; Farnum and Wilsman, 1989). In support of this, the ultrastructure of terminal hypertrophic chondrocytes located in the mineralized zone indicated intact cells, such making it difficult to reconcile that these cells are dying (Holtrop, 1972; Hunziker et al., 1984). Moreover, mitotic divisions have been observed to occur in hypertrophic chondrocytes located within the mineralized area (Crelin and Koch, 1967). This corroborates our observations that CDOPs at the chondro-osseous junction are mitotically active and take up BrdU. Although they express some stem cell markers such as CD34, c-Myc, Sox2 and Sca1, we do not have evidence yet that CDOPs are actually stem-like cells. But in light of the fact that more than half of the YFP^+^ cells in the spongiosa of E18.5 humeri were Osx negative, it appears that a fraction of CDOP cells also enters non-osteogenic cell lineages.

The question remained how mature hypertrophic chondrocytes of 10–20 µm diameter in the fully hydrated form are able to shrink to the “condensed” form (Farnum and Wilsman, 1989) with a similar size as CDOPs (4–6 µm). Although currently still speculative, one likely mechanism would be autophagy, an evolutionary conserved intracellular degradation process in all eukaryotic cells that acts as a cell protective mechanism under stress conditions and thus may prevent apoptosis (Yang and Klionsky, 2010). By sequestering dysfunctional organelles, proteins and membranes into double-membrane vesicles called autophagosomes, which later fuse with lysosomes, cells generate energy and remain viable and complete their life cycle (Kimura et al., 2007; Srinivas et al., 2009b; Yang and Klionsky, 2009). Since regulation of autophagy and apoptosis are intimately connected and sometimes controlled by the same regulators (Thorburn, 2008), the nature of local signals deciding on either process for hypertrophic chondrocytes in different locations has yet to be identified. Another mechanism that may contribute to the shrinkage is the reversal of the swelling process that is in part contributing to the immense size increase of hypertrophic chondrocytes – a process that is not yet understood either (Cooper et al., 2013).

In conclusion, our findings demonstrate that late hypertrophic chondrocytes are able to give rise to a distinct population of osteoblasts, which contribute to trabecular, endosteal and cortical bone formation. We provide evidence that they originate from small cells called CDOPs localized in the lowest hypertrophic zone, which may be related to the “condensed cells” identified ultrastructurally by Farnum and Wilsman (Farnum and Wilsman, 1987; Farnum and Wilsman 1989) in the next to the lowest lacunae of hypertrophic growth plate cartilage. They are mitotically active and are able to differentiate into osteoblasts *in vivo* and *in vitro*. This implies a novel, active role for hypertrophic chondrocytes by giving rise to an osteogenic progeny and challenges the view that chondrocyte differentiation is inevitably terminated in the late hypertrophic zone of the growth plate.

## Supplementary Material

Supplementary Material
