# Supplementary material for: Dual pathways to endochondral osteoblasts: a novel chondrocyte-derived osteoprogenitor cell identified in hypertrophic cartilage
Source: Biol Open. 2015 Apr 16;4(5):608–21. doi: 10.1242/bio.201411031 (PMC4434812; doi:10.1242/bio.201411031)
Supplement: Supplementary Material [file supp_4_5_608__index.html]

Dual pathways to endochondral osteoblasts: a novel chondrocyte-derived osteoprogenitor cell identified in hypertrophic cartilage — Supplementary Material 

# Dual pathways to endochondral osteoblasts: a novel chondrocyte-derived osteoprogenitor cell identified in hypertrophic cartilage

## bio.201411031 Supplementary Material

**Files in this Data Supplement:**

- Supplementary Material
