# Supplementary material for: Dual pathways to endochondral osteoblasts: a novel chondrocyte-derived osteoprogenitor cell identified in hypertrophic cartilage
Source: Biol Open. 2015 Apr 16;4(5):608–21. doi: 10.1242/bio.201411031 (PMC4434812; doi:10.1242/bio.201411031)
Supplement: Supplementary Material [file supp_bio.201411031_Biology_Open-2015-Park-bio_201411031_supp.pdf]

Biology Open (2015) 000, 1–14 doi:10.1242/bio.201411031

Supplementary Material
Jung Park et al. doi: 10.1242/bio.201411031

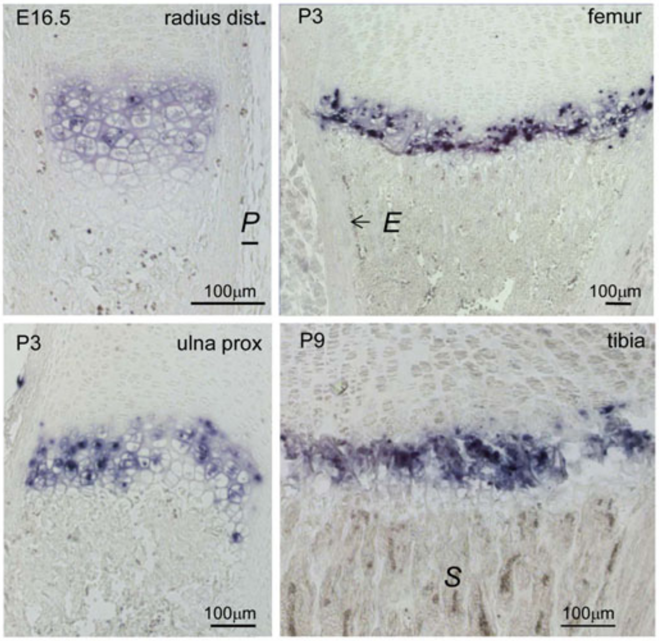

Fig. S1. Specific expression of BACCol10Cre in hypertrophic chondrocytes. *In situ* hybridization on skeletal elements from *BACCol10Cre* mice confirms exclusive *Cre* expression in hypertrophic chondrocytes and absence of signals from trabecular bone in the spongiosa ‘S’. P: periosteum, E: endosteum.

Biology Open (2015) 000, 1–14 doi:10.1242/bio.201411031

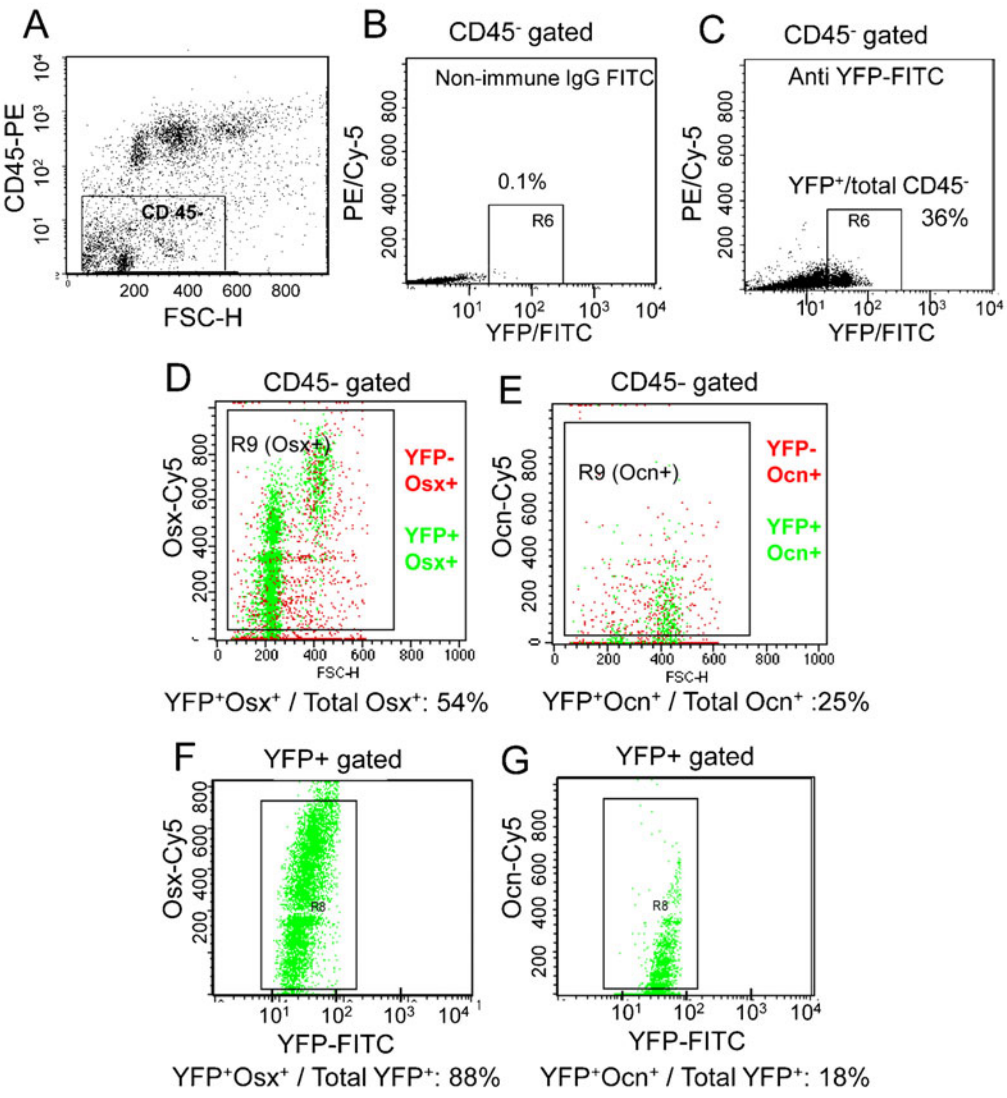

Fig. S2. FACS Analysis of endosteal cells isolated from long bones of Col10CreYFP^{+} mice. Spongiosa and bone marrow was scraped out from the bone shafts of P20 Col10Cre YFP^{+} tibiae and femora after removal of epiphyses including growth plates. Shafts were crushed and plated on collagen coated 6 cm dishes in MEM/10% FCS. Cell outgrowth was allowed for 1 week, then cells were dissociated with collagenase, briefly fixed in 4% paraformaldehyde and washed. FACS analysis was performed in a Becton-Dickinson Calibur Analyzer. (A) Cells were stained with goat FITC-anti GFP (Abcam) and rabbit anti CD45-PE; only CD45 negative cells were gated. (B) CD45 negative cells stained with non-immune IFG-FITC only. (C) Staining with anti YFP shows that 36% of all CD45^{-} cells, were YFP^{+} (in R6). (D) Co-staining with anti YFP and rabbit anti Osx-Cy5: 54% of all Osx^{+} endosteal derived cells are YFP^{+}. (E) Co-staining with anti YFP and rabbit anti osteocalcin (Ocn) –Cy5: 25% of all Ocn^{+} endosteal derived cells are YFP^{+}. Note: the two YFP^{+} size populations visible in D and E probably reflect smaller osteoprogenitor cells and mature osteoblasts, respectively. (F,G) Gating for YFP^{+} cells shows that of all YFP^{+} cells 88% are Osx^{+} and 18% Ocn^{+}.

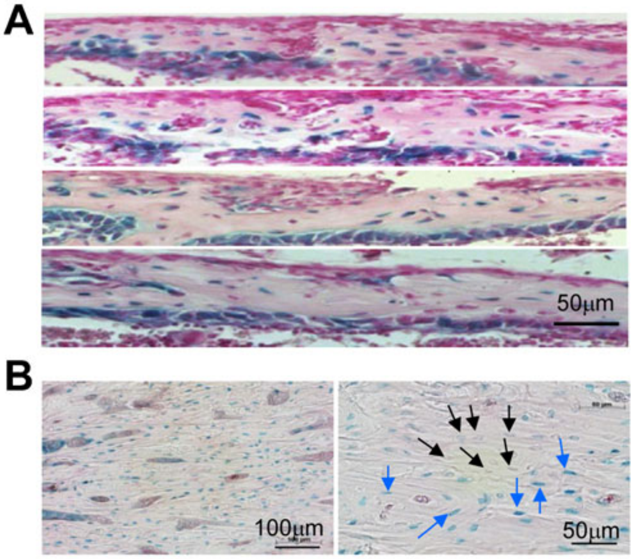

Fig. S3. Chondrocyte–derived (LacZ$^+$) osteocytes and periosteum-derived osteocytes (LacZ$^-$) contribute about equally to cortical bone in P17 to P35 long bones. (A) Paraffin section of cortical bone shaft from P17 *Col10Cre; R26R* tibiae were stained with X-gal and counterstained with nuclear fast red. Ratios of blue osteocytes/total osteocytes were 40,87%, SD±16.5% as calculated from counting 13 fields as depicted (total 554 cells). Only bone matrix encapsulated osteocytes, no osteoblasts nor endosteal cells were included for counting. (B) *LacZ*$^+$ osteocytes (blue arrows) in cortical bone shafts of P35 tibiae constitute about 50% of all cortical osteocytes. Black arrows: LacZ$^-$ osteocytes.

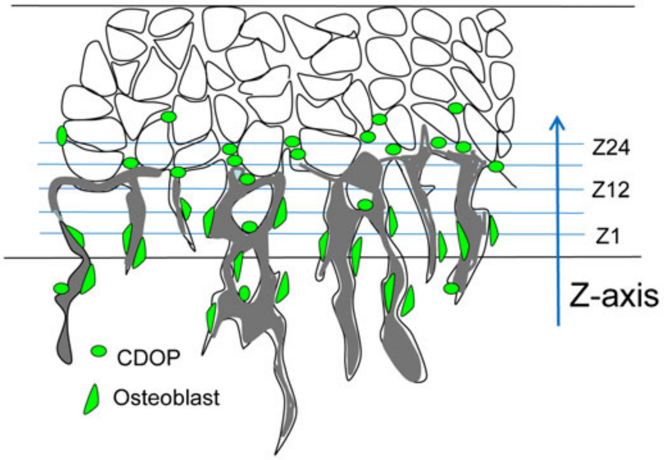

Fig. S4. Analysis of the lower hypertrophic zone of the chondro-osseous junction by confocal microscopy. Chondrocyte derived osteoprogenitor cells (CDOPs) are located predominantly in the lowest layer of hypertrophic cartilage and at the interface between hypertrophic cartilage and spongiosa. The arrow indicates the direction of the z-axis. 24 sections were analyzed per growth plate.

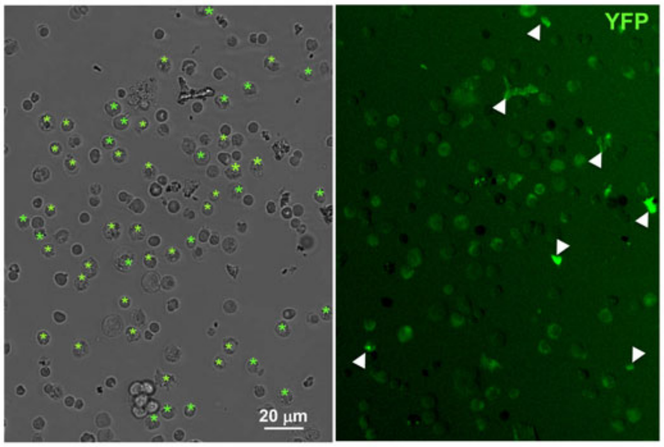

Fig. S5. YFP fluorescence of fraction 1 cells prepared from Col10CreYFP$^+$ growth plates. The average rate of YFP$^+$ cells in fraction 1 was 22±8.9% as determined after counting eight fields in four separate wells. Fraction 2 contained 34±9.4% YFP$^+$ cells (not shown). Fluorescent cell debris was not counted. In the left bright-field image the YFP$^+$ cells are marked with an asterisk. Some of the bright spots in the YFP image on the right side were not counted, as they were considered to be debris based on their appearance (e.g. white arrowheads). Analyzed with a Zeiss Apotome microscope.

Biology Open (2015) 000, 1–14 doi:10.1242/bio.201411031

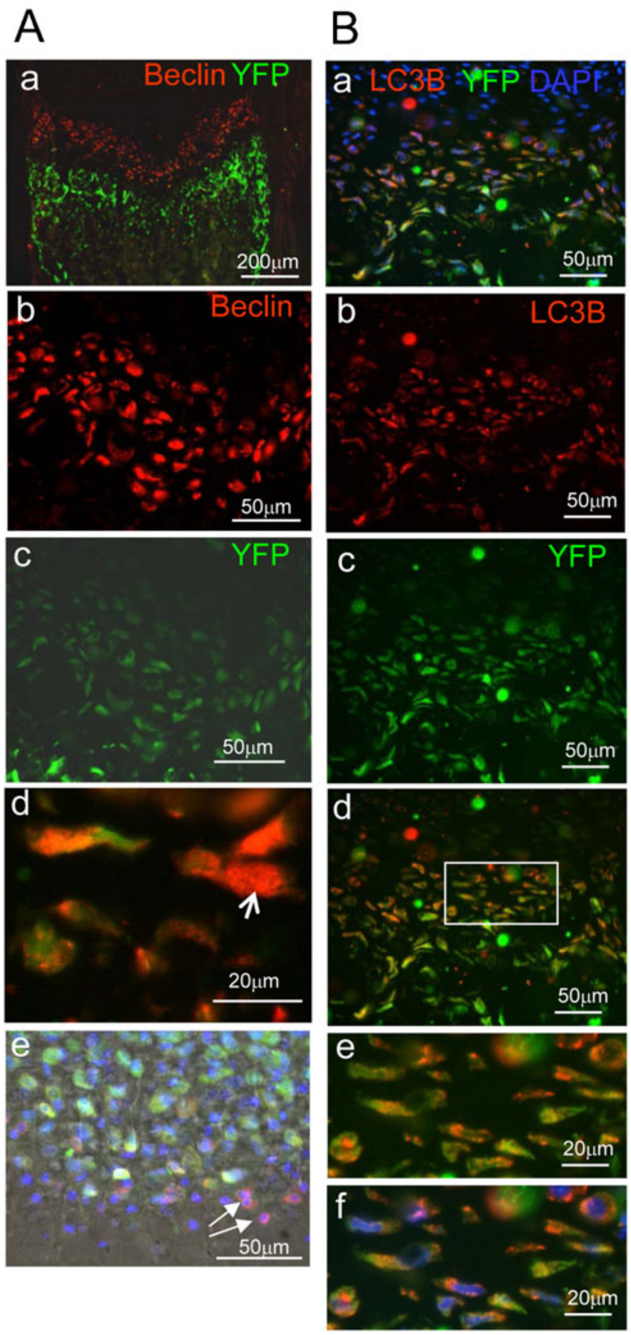

Fig. S6. Expression of beclin and LC3B indicates autophagy in hypertrophic chondrocytes. (A) Anti beclin-1 (red) stains prehypertrophic and hypertrophic chondrocytes in the proximal growth plate of a P7 Col10CreYFP tibia (a), coinciding with the expression of YFP (a–c). (d) Anti beclin labels autophagosomes which appear as punctate structures (arrow in d); (e) confocal microscopy reveals small, CDOP-like beclin$^+$ cells in the lower hypertrophic zone. (Ba–f) Prehypertrophic and hypertrophic chondrocytes also express LC3B (red) which is required for the expansion of autophagosomes. (e,f) Higher magnification of insert in d. P7, tibia growth plate. Antibodies: Rabbit anti-beclin 1, Abcam 16998; Rabbit anti-LC3B, Cell Signalling #2775.

Biology Open

Biology Open (2015) 000, 1–14 doi:10.1242/bio.201411031

Table S1. Primer sequences

Primers for *in situ* hybridization probes	
Cre-reverse	5′-AAAATTTGCCTGCATTACCG-3′
Cre-forward	5′-ATTCTCCCACCGTCAGTACG-3′
YFP-reverse	5′-GACGTAAACGGCCACAAGAT-3′
YFP-forward	5′-GAACTCCAGCAGGACCATG-3′
Primers for genotyping	
Cre:P1 (Col10a1Int)	5′-TTTAGAGCATTATTTCAAGGCAGTTTCCA-3′
Cre: P5 (Cre rev)	5′-AGGCAAATTTTGGTGTACGG-3′
YFP:R26YFP 4982	5′-AAGACCGCGAAGAGTTTGGTC-3′
YFP:R26 wt 0883	5′-AAAGTCGCTCTGAGTTGTTAT-3′
YFP:R26 wt 0316	5′-GGAGCGGGAGAAATGGATATG-3′
Primers for RT-PCR and qRT-PCR	
runx2-for	5′-AGGTTGGAGGCACACATAGG-3′
runx2-rev	5′-ATACCCCCTCGCTCTCTGTT-3′
Ocn-for	5′-CAAGTCCCACACAGCAGCTT-3′
Ocn-rev	5′-CTTTCCCCAGGGTTGTTGAG-3′
Cyclophilin-for	5′-CCACCGTGTTCTTCGACAT-3′
Cyclophilin-rev	5′-CAGTGCTCAG AGCTCGAAAG-3′
Col1a1-for	5′-CAGGGGGACCAGGAGGACCAGGAAGT-3′
Col1a1-rev	5′-CCCCACCCCAGCCGCAAAGAGT-3′
Osx-for	5′-ATGGCGTCCTCTCTGCTTG-3′
Osx-rev	5′-CTTTCCCCAGGGTTGTTGAG-3′

Biology Open
